# Early Childhood Emotional, Relational, and Stress‐Related Outcomes Following Infant Cardiac Surgery

**DOI:** 10.1002/dev.70200

**Published:** 2026-09-14

**Authors:** Tamera A. Clancy, Carolina de Weerth, Frank Muscara, Brigid Jordan

**Affiliations:** ^1^ Department of Paediatrics University of Melbourne Melbourne Victoria Australia; ^2^ Brain and Mind Murdoch Children's Research Institute Melbourne Victoria Australia; ^3^ Donders Institute for Brain, Cognition and Behaviour Radboud University Medical Center Nijmegen The Netherlands; ^4^ The Royal Children's Hospital Melbourne Victoria Australia

**Keywords:** cardiac, cortisol, preschool, relationship, stress response

## Abstract

Children with congenital heart disease (CHD) experience significant early‐life stress through medical interventions and hospitalization, shaping physiological and psychological development. This study examined emotional and behavioral regulation and salivary cortisol responses in preschool‐aged children who underwent cardiac surgery in infancy, and explored the parent–child relationship as a source of co‐regulation and buffering support. In this prospective cohort, 22 preschoolers with CHD and their parents participated in a hospital‐based laboratory visit. Child salivary cortisol was collected at three time points. Observed parent–child interactions were coded using the Emotional Availability Scales, and parent‐reported Child Behavior Checklist (CBCL) scores assessed emotional and behavioral regulation. Mixed‐effects models examined associations between cortisol response, CBCL scores, and relationship quality. Significant individual variability in cortisol response was observed. Lower cortisol levels were associated with greater emotional and behavioral difficulties and higher parent‐reported traumatic stress at the time of their child's surgery. Longer hospital stays predicted blunted cortisol responses. Children in lower quality relationships displayed flatter cortisol profiles. Findings highlight the impact of early medical stress on stress physiology and suggest emotionally available relationships may buffer HPA axis dysregulation. Results identify potential intervention targets to support psychosocial development and parent–child relationships and contribute to the emerging use of stress biomarkers in CHD research.

## 1 Introduction

Cardiac surgery for congenital heart disease (CHD) early in life, though medically necessary, is an inherently stressful experience (Wernovsky 2006). Infants face repeated invasive procedures, frequent handling, pain, and separation from parents (Lerwick 2016) during a critical period of rapid neurobiological development when stress sensitivity is heightened (Heim and Nemeroff 2001). Exposure to these potentially traumatic medical events and challenges associated with parenting a child with CHD can strain the developing parent–child relationship (Jordan et al. 2014), with up to 80% of parents reporting elevated psychological distress (Woolf‐King et al. 2017). Children with CHD are at increased risk for emotional and behavioral regulation difficulties (Karsdorp et al. 2007), yet the mechanisms underlying these developmental outcomes remain poorly understood (Cassedy et al. 2023). Beyond direct threats to physical integrity, stressors that challenge social relationships, particularly those that are uncontrollable or unpredictable, are especially potent in shaping the stress response (Dickerson and Kemeny 2004; Koss and Gunnar 2018). In the context of early cardiac surgery for CHD, children and families are often exposed to stressors that are both uncontrollable (e.g., medical procedures) and unpredictable (e.g., clinical course and recovery).

Early‐life stress is known to shape the developing hypothalamic–pituitary–adrenal (HPA) axis (Gunnar et al. 2009), with potential long‐term consequences for stress regulation (Champagne et al. 2009; Loman and Gunnar 2010; Shonkoff and Garner 2012). Infants exposed to painful medical procedures demonstrate elevated cortisol levels in the first 2 years of life, suggesting that early medical experiences may alter the set point of arousal (Grunau et al. 2004). In children with CHD, McGauran et al. (2017) observed differences in cortisol responses to echocardiograms at 3–5 years of age between those who underwent surgery before 6 months of age and those who underwent surgery later. Recent research using hair cortisol, a marker of cumulative HPA axis activity, has demonstrated elevated long‐term cortisol levels in adolescents with complex CHD compared to healthy controls (von Werdt et al. 2024). These findings are consistent with evidence from other high‐risk pediatric populations showing that early adversity can influence both diurnal and stress‐reactive cortisol patterns (Grunau 2013; Hunter et al. 2011). Furthermore, altered cortisol regulation has been linked to poorer developmental and mental health outcomes (Koss and Gunnar 2018).

Cortisol, the end product of HPA axis activation, is a key biomarker of stress regulation and an integral part of the adaptive stress response in children (Koss and Gunnar 2018), supporting energy mobilization, modulating inflammation, and aiding stress recovery (Gunnar and Donzella 2002). However, early adversity can have prolonged influence on cortisol patterns, reflecting either adaptive regulation or maladaptive dysregulation (Gunnar and Quevedo 2007). Dysregulated cortisol levels can manifest as elevated (hyperactivity) or blunted (hypoactivity), reflecting adaptations to repeated stress exposure (Lupien et al. 2009; McEwen 1998). Although use of cortisol as a stress biomarker in pediatric research is well‐established (Slopen et al. 2014), few studies have examined its relevance in CHD populations. Pioneering research using salivary cortisol has indicated its potential as a feasible, noninvasive stress biomarker in children with CHD (Brandlistuen et al. 2011; McGauran et al. 2017; Naguib et al. 2013).

Early medical adversity can undermine the capacity of relationships to provide stress buffering and regulation benefits (Koss and Gunnar 2018). Yet, importantly, this aspect of the CHD experience is potentially modifiable. For children, parents are potent regulators of HPA axis reactivity, particularly through promoting positive outcomes by buffering the impact of stressful experiences (Albers et al. 2008; Hostinar and Gunnar 2013; Hostinar et al. 2015). Sensitive caregiving influences HPA axis reactivity (Lupien et al. 2009), with emotionally available relationships helping to regulate children's cortisol levels and stress (Philbrook et al. 2014; Senehi et al. 2021).

A growing body of work suggests that children are highly attuned to their caregivers’ emotional and physiological states, particularly in the context of heightened parental stress, with evidence of coordinated patterns of stress responding within parent–child dyads, reflecting processes described as stress contagion and adrenocortical attunement (Hibel et al. 2015; Waters et al. 2014). In the context of CHD, where parents experience heightened and ongoing stress related to their child's medical condition and treatment, these processes may represent an additional pathway through which early experiences shape children's stress regulation.

Despite the known psychosocial risks for children with CHD (Clancy et al. 2020), few studies have examined the intersection of physiological stress markers, relational context, and psychosocial outcomes. The present study constitutes an exploratory step toward addressing this gap by investigating cortisol response as a variable of interest in relation to preschool emotional and behavioral outcomes following infant cardiac surgery. McGauran et al. (2017) found that children who underwent cardiac surgery before 6 months of age exhibited elevated cortisol responses to an echocardiogram, providing compelling evidence of altered stress physiology in response to a specific medical procedure. Extending this line of inquiry, we examined cortisol response during a laboratory visit within the hospital setting to assess whether the environment itself might elicit similar HPA axis activation in children with early cardiac surgery history. We also examined the buffering role of the parent–child relationship in modulating preschool outcomes. Many parents of children with CHD experience high levels of trauma related to their child's diagnosis, surgery, and hospitalization (Sood et al. 2021), which may compromise their emotional availability and capacity to buffer their child's stress. While the developmental trajectory of children with CHD is multifaceted and complex, early stressors associated with surgery and hospitalization may represent risk factors that, while not entirely avoidable, can be influenced through early relational and psychosocial support. This study aligns with calls for more integrative, developmentally informed CHD research (Cassidy and Sanz 2023), building an evidence base using stress biomarkers in research with children with CHD (Sood et al. 2021).

## 2 Methods

This study is part of *the Heart Supports Study*, a longitudinal, prospective cohort study of preschool‐aged children with CHD who underwent cardiac surgery within the first 6 months of life. The primary aim is to examine patterns of cortisol response in children with CHD during a structured laboratory visit in a hospital setting.

Hypotheses are as follows:

‐ Preschoolers with CHD will exhibit a measurable cortisol response across a laboratory visit in a hospital environment.
‐ The child's cortisol response will be associated with length of hospital stay, parental traumatic stress at time of infant cardiac surgery admission, and emotional and behavioral regulation at preschool age.
‐ Parent–child relationship quality will be associated with cortisol response.

Given the limited literature, these hypotheses are exploratory, generating initial evidence to inform future research.

### 2.1 Ethical Considerations

The Human Research and Ethics Committee (HREC) at the Royal Children's Hospital (RCH), Melbourne, Australia, approved all methods used in this study. All parents gave written informed consent on behalf of their child and themselves, prior to participation (HREC #34174).

### 2.2 Design

This is a prospective, cohort study with a longitudinal design, utilizing quantitative research methods including standardized, clinician‐rated observational measures, salivary cortisol, parent report questionnaires, and medical data. Time 1 refers to early infancy at the time of discharge from hospital following cardiac surgery for CHD, and Time 2 refers to our follow‐up study of children at preschool age (3–4 years old).

### 2.3 Participants

Recruitment of participants was from a longitudinal study examining the impact of early cardiac surgery on infants and parental adjustment, *The Heart Supports Study*. Infants were eligible for that study if they had open or closed cardiac surgery for correction of a CHD within the first 6 months of life at the RCH from December 2014 to December 2015. CHDs are commonly classified as cyanotic or acyanotic based on their physiological impact (Hoffman and Kaplan 2002). Cyanotic CHD involves reduced oxygenation of circulating blood, often reflecting greater clinical severity, whereas acyanotic CHD typically does not impair systemic oxygenation to the same extent. For the present study, classification was determined by the consulting cardiac surgeon. Children underwent cardiac surgery with standard care requiring admission to the cardiac intensive care unit, followed by cardiology ward admission for postoperative care. Fifty‐three infants and their parents participated at Time 1. At Time 2 (January–August 2019), 33 of 53 (62.26%) preschooler–parent dyads of the Heart Supports cohort participated. Children were between 3 and 4 years of age. Of those dyads, 22 participated in the in‐person aspects of data collection and are the focus of this paper. Eleven were unable to attend in person for data collection (due to geographical distance and one child's compromised health). Twenty families were lost to follow‐up (unable to establish contact [*n* = 10], child died [*n* = 1], and chose not to participate in the present study [*n* = 9]). Valid cortisol samples were available for 20 preschoolers at Sample 1, 19 at Sample 2, and 20 at Sample 3. To be included in the mixed‐effects regression analyses, participants were required to have valid cortisol data across all three sampling time points. As a result, three participants were excluded due to insufficient saliva volume, yielding a final analytic sample of 19 participants. Of the 33 dyads who completed Time 2, participants with valid cortisol data did not differ significantly from those without valid cortisol data on available demographic and clinical variables, including child age at Time 2 (*t*(31) = 0.66, *p* = 0.51), length of hospital stay (*t*(31) = 0.95, *p* = 0.35), child sex (Fisher's exact *p* = 0.30), CHD type (Fisher's exact *p* = 0.28), timing of diagnosis (Fisher's exact *p* = 0.51), or parent education (Fisher's exact *p* = 0.87).

### 2.4 Procedure

At Time 1, parents completed the *Heart Supports Study: Infant and Family Wellbeing* questionnaire, 6 weeks after their infant (*M*_age_ = 5 months, SD = 1.8) was discharged from hospital following cardiac surgery.

At Time 2, participants and their parent(s) attended one laboratory visit for data collection purposes (duration 1–1.5 h) at the RCH, the facility in which their cardiac surgery, hospitalization, and outpatient appointments occurred. To minimize diurnal variation in cortisol levels, laboratory sessions were scheduled between 12:30 p.m. and 2:30 p.m. The laboratory visit followed a standardized schedule including an initial familiarization period, collection of saliva samples at planned intervals across the visit, a series of structured play‐based tasks, and a video‐recorded parent–child free‐play interaction. Parents also completed the Time 2 study questionnaire, which assessed sociodemographic and preschooler medical information and child emotional and behavioral functioning, as described below. A summary of the laboratory visit schedule and saliva sampling timeline is provided in Table S1.

### 2.5 Measures

#### 2.5.1 Time 1

The *Heart Supports Study* *: Infant and Family Wellbeing* questionnaire comprised standardized and qualitative measures, recording infant medical history, sociodemographic characteristics, psychological well‐being of the infant and parent, and support services. To address the aims of this study, measures from Time 1 were chosen to assess the key theoretical constructs of interest, specifically early experience of stress and early opportunities for co‐regulation and buffering support following infant cardiac surgery, specifically:

*Length of hospital stay* (days) at the time of infant cardiac surgery was used as a proxy for the degree of stress experienced during hospitalization. This allowed the investigation of whether length of stay was related to stress response (measured by cortisol concentrations) during a laboratory visit in the same hospital setting at preschool age.

*Parental traumatic stress* was measured using the Posttraumatic Stress Disorder Checklist for DSM‐5 (PCL‐5) (Weathers et al. 2013), a 20‐item self‐report questionnaire. Parents indicated how much they were bothered by symptoms in the past month, on a 5‐point Likert scale ranging from 0 = *not at all* to 4 = *extremely*. The PCL‐5 assesses four PTSD symptom clusters: intrusion, avoidance, negative alterations in cognition and mood, and hyperarousal. Total scores range from 0 to 80, with higher scores indicating greater symptom severity. The PCL‐5 has good psychometric properties, including high internal consistency (*α* = 0.94), test–retest reliability (*r* = 0.82), and concurrent validity (*r* = 0.74–0.85) (Blevins et al. 2015).

#### 2.5.2 Time 2

Parents completed the *Heart Support Study: Life as a Preschooler Study* questionnaire, which included sociodemographic and preschooler medical information and a standardized measure of child emotional and behavioral regulation.

*Emotional and behavioral regulation* was measured using the Child Behavior Checklist: 1.5–5 years (CBCL) (Achenbach and Rescorla 2000), a 99‐item norm‐referenced parent report questionnaire of child behavior in these domains: emotional reactivity, anxiety/depression, somatic complaints, withdrawal, sleep problems, attention problems, and aggressive behavior. Frequency of behaviors is rated over the previous 2 months from 0 = *not true* to 2 = *very true or often true*. Externalizing scale (e.g., aggression, defiance, and conduct problem behaviors), internalizing scale (e.g., anxiety, depression, somatic complaints, and withdrawal behaviors), and total raw scores are calculated and transformed into standardized *T* scores (*M* = 50, SD = 10). The CBCL defines *T*‐scores ≥60 as representing a borderline/clinical problem and a *T*‐score ≥64 as representing a clinical problem. Alpha coefficients for internal consistency have been reported as 0.89 for the internalizing subscale, 0.93 for the externalizing subscale, and 0.95 for the total score (Achenbach and Rescorla 2000). As a normative Australian sample has not been generated for this scale, published normative data for a US sample were used for comparison (Achenbach and Rescorla 2000).

*Physiological Measurement of Stress Response* Cortisol response, defined as changes in salivary cortisol concentration, was measured across a structured laboratory visit conducted within the hospital setting where the child's cardiac surgery, hospitalization, and outpatient appointments occurred. This approach was intended to examine patterns of cortisol responding within a clinically meaningful context characterized by repeated early medical exposure. The first saliva sample (baseline: S1) was taken after arrival at the laboratory. The child then interacted with an unfamiliar researcher and participated in an established battery of age‐appropriate, structured behavioral tasks for approximately 25 min (Goldsmith and Rothbart 1996; Kochanska et al. 1996). These tasks were selected because they have been used in research examining similar constructs (Kertes et al. 2009; Spinrad et al. 2007). A second saliva sample (S2) was taken approximately 25–30 min after S1 to capture cortisol concentrations during the first period in the laboratory. Children then participated in a play‐based interaction with their parent (described below), followed by a third and final saliva sample (S3) collected approximately 50–60 min after S1. The timing of samples was determined on the basis of findings that salivary cortisol concentrations reflect the degree of stress experienced in the prior 20–40 min (Gunnar et al. 2009; Hanrahan et al. 2006; Kirschbaum and Hellhammer 1994).

Parents were asked not to allow their child to eat or drink in the 30 min prior to the visit to minimize error in cortisol assay and reported on medication intake and recent illness (DePasquale et al. 2019; Magnano et al. 1989; Schwartz et al. 1998). Parents assisted their child to insert a cotton swab (SalivaBio Children's Oral Swabs; 125 mm synthetic swab) into their mouth for passive saliva collection. The swabs were stored in capped swab storage tubes and immediately placed on ice and transported to −30°C freezers for storage in the Murdoch Children's Research Institute laboratory. All saliva samples were transported to the Division of Laboratories, Pharmacy and Biomedical Genetics, University Medical Centre (UMC), Utrecht, The Netherlands, and stored at −30°C before and after analysis by the UMC laboratory. Cortisol in saliva was measured without extraction using an in‐house competitive radioimmunoassay employing a polyclonal anti‐cortisol antibody (K7348). [1,2–3 H(N)]‐Hydrocortisone (PerkinElmer NET396250UC) was used as a tracer. The lower limit of detection was 1.0 nmol/L, and interassay variation was <8% at 1.0–31 nmol/L (*n* = 22). Samples were treated as “missing” if they contained too small a volume to analyze.

*Quality of the parent–child relationship* was measured using the Emotional Availability (*EA*) Scales, 4th Edition—Infancy/Early Childhood (Biringen 2008). The EA Scales are a global measure of the emotional availability of the parent–child relationship and focus on child as well as parent qualities. Children and their parents were instructed to engage in free play for 20 min with a standardized set of toys provided. The researcher was not present during the interaction. The EA includes four adult (Sensitivity, Structuring, Nonintrusiveness, Nonhostility) and two child (Responsiveness, Involvement) dimensions. The videoed interaction was coded according to Biringen (2000) by two coders trained and certified as reliable by the developer of the EA coding system (Biringen 2008). Each dimension was rated globally across the full 20‐min interaction and scored using a 7‐point scale, from 1 (*low*) to 7 (*high and the optimal score*) (Biringen 2008; Biringen et al. 2022). Interrater reliability estimates were calculated as intraclass correlation coefficients for the direct scores on each EA scale dimension (sensitivity 0.95, structuring 0.92, nonintrusiveness 1.0, nonhostility 0.95, child responsiveness 1.0, and child involvement 0.95). The construct validity of the EA is well‐established, and EA has also demonstrated short‐term and long‐term stability (Biringen et al. 2014). Brief descriptions of each EA dimension are provided in Table S2.

Next, an EA Zone (EA‐Z) score was calculated on a dimensional 1–100 global rating scale as a summary of the EA scales coded above and was used to categorize EA into four distinct zones, including Emotionally Available, Complicated, Detached, and Disturbed/Traumatized (Senehi et al. 2021). For the relationship to be scored in the Emotionally Available category (81–100), both parent and child are available and responsive to each other. In contrast, dyads in the Complicated range (61–80) indicate incongruity in emotional availability, and the Detached category indicates an emotional disconnection, but basic needs are met (41–60). The Disturbed/Traumatized category (0–40) indicates an extreme lack of parental sensitivity and child responsiveness (Biringen 2008; Espinet et al. 2013). After scoring, EA‐Z scores were dichotomized, consistent with the methodology outlined by Senehi et al. (2021), to create two groups of high‐quality interaction (EA‐Z = 1) (scores ≥81) and low‐quality interaction (EA‐Z = 0) (scores ≤80). These dichotomized scores were used in mixed‐effects regression analyses.

### 2.6 Statistical Analysis

Data were analyzed using STATA Statistical Software Release 18 (StataCorp 2023). As this study utilized an existing cohort with a fixed sample size, a formal a priori power analysis was not conducted. Accordingly, analyses were considered exploratory. Demographic characteristics were described using means (SD) or frequencies (%), and descriptive statistics were examined for all variables.

Cortisol concentrations were approximately normally distributed based on visual inspection and Shapiro–Wilk testing. Sensitivity analyses using log‐transformed cortisol values yielded substantively unchanged findings; therefore, raw cortisol values were retained for analysis and interpretation. However, some variables, including parental traumatic stress and length of hospital stay, exhibited skewed distributions. Given the small sample size and deviations from normality, nonparametric tests (Spearman's rank‐order correlations and Mann–Whitney *U* tests) were used to examine initial bivariate associations between variables. To obtain robust estimates, bootstrapping with 1000 replications was applied to calculate bias‐corrected 95% confidence intervals (CIs) and *p*‐values.

Mixed‐effects regression models were used to account for the hierarchical structure of the data, with cortisol concentrations at each sampling time point used as the dependent variable. A random intercept model was specified, with participant‐level variability modeled as a random effect to account for individual differences in baseline cortisol concentrations. Time (cortisol measurement points) was entered as a fixed effect for all models. Hypothesis 1 was tested using a mixed‐effects regression model with time as a fixed effect to assess overall change in cortisol concentrations during the laboratory visit. To examine bivariate associations between cortisol and study variables (Hypothesis 2), average cortisol concentrations across the three sampling time points were calculated for each participant, and nonparametric analyses (Spearman's rank‐order correlations and Mann–Whitney *U* tests) were conducted. Length of hospital stay and parental traumatic stress were treated as predictors in the model, with cortisol concentrations as the outcome. Averaging cortisol values was used to provide an estimate of overall cortisol output across the laboratory visit, given the small sample size. For Hypothesis 3, relationship quality was treated as a fixed effect to examine its main effect and interaction with time on cortisol concentrations. To reduce model complexity in the context of the small sample, length of hospital stay and parental traumatic stress were examined in separate covariate‐adjusted models.

Model assumptions, including normality of residuals and homoscedasticity, were checked and robust standard errors were applied where necessary. Length of hospital stay was skewed (Shapiro–Wilk *W* = 0.77, *p* < 0.001), including one participant with a prolonged hospitalization of 110 days. A log transformation was explored to address the skewed distribution. A sensitivity analysis excluding this participant showed that the association between length of hospital stay and cortisol remained negative (*B* = −0.05), although it was no longer statistically significant (*p* = 0.07). As the direction of the association was consistent and the participant with the 110‐day stay represented a clinically valid observation, raw length of stay values were retained to allow for more direct interpretation of findings in relation to hospital stay duration (Field 2013). A *p*‐value of less than 0.05 was considered significant.

## 3 Results

### 3.1 Participant Characteristics

Participants were 22 children (*M*_age_ = 47.59 months, SD = 3.22) and their parents (*M*_age_ = 35.96 years, SD = 3.75) (Table 1). Most parents were female (94%), as were 59% of children, and 66% of parents had completed tertiary education. Cardiac condition was classified as cyanotic for 63% of children in this study, with 36% acyanotic. We sought to ensure greater sensitivity to the medical/physical status of the infant at the time of surgery by including a measure of length of hospital stay (days). At the time of their cardiac surgery, children in this follow‐up study stayed in hospital between 5 and 110 days, with a median length of stay of 18 days and an interquartile range of 14–35 days. Overall, preschoolers in this cohort demonstrated levels of emotional and behavioral functioning comparable to normative data. One‐sample *t*‐tests indicated no significant differences between this cohort and normative means across Internalizing (*t*(21) = 0.59, *p* > 0.05, *d* = 0.12), Externalizing (*t*(21) = −0.46, *p* > 0.05, *d* = −0.10), and Total Problems scores (*t*(21) = 0.33, *p* > 0.05, *d* = 0.07).

**Hypothesis 1**. *Preschoolers with CHD will exhibit a measurable cortisol response to a laboratory visit in a hospital environment*.

**TABLE 1:** Descriptive statistics and exploratory bivariate associations between average cortisol concentrations and study variables.

Variable, *n* = 19	Mean (SD); range or *n* (%)	Spearman's rho	*p* value	95% CI (bootstrapped)
Cortisol levels (nmol/L^a^)				
Sample 1	5.27 (2.08); 1.3–9.5	**—**	**—**	**—**
Sample 2	4.91 (2.14); 1.0–8.8	**—**	**—**	**—**
Sample 3	4.67 (1.85); 1.8–8.0	**—**	**—**	**—**
Child age (months)	47.59 (3.22); 42–54	0.21	0.42	−0.29, −0.72
Child sex (female)	12 (67%)	−0.45	0.02^*^	−0.82, −0.09
Time 1 variables				
Confounders				
Length of stay (days) (median, IQR)^b^	18 (14–35)	−0.33	0.01^**^	−0.58, −0.07
Parental traumatic stress^b^	10.54 (8.63); 0–30	−0.23	0.04^*^	−0.46, −0.002
Time 2 variables				
Emotional and behavioral regulation^c^				
Total problems	50.67 (9.65; 34–66)	−0.37	0.001^**^	−0.58, −0.16
Internalizing	51.23 (9.87; 37–65)	−0.40	0.001^**^	−0.62, −0.18
Externalizing	49.05 (9.73; 32–67)	−0.28	0.03^*^	−0.54, −0.03
Parent–child relationship quality^d^		*z* = −2.06^e^	0.02^*^	−3.85, −0.27
High quality	12 (55%)	**—**	**—**	**—**
Low quality	10 (45%)	**—**	**—**	**—**

^a^ nmol/L = nanomoles per litre.

^b^ Measured at Time 1.

^c^ As measured by the CBCL scales.

^d^ As measured by EA‐Z score; high quality = 81–100; low quality = 0–80.

^e^ Mann–Whitney *U* test was used as relationship quality is a dichotomous variable. Bootstrapping (1000) replications was applied to obtain robust *p*‐values and confidence intervals, given the small sample size.

*p < 0.05; **p ≤ 0.001.

**FIGURE 1:**
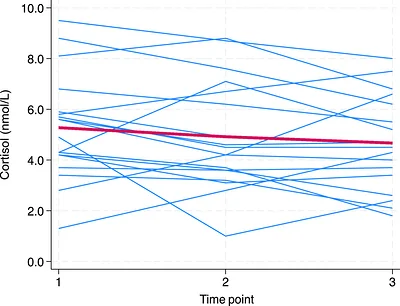
Individual cortisol trajectories during the laboratory visit and mean cortisol concentrations at each sample time point. Individual salivary cortisol concentrations are shown across the three sample time points. Blue lines represent individual child trajectories, and the red line represents the mean cortisol concentrations at each time point. Cortisol concentrations are reported in nanomoles per liter (nmol/L).

We examined change in child cortisol concentrations over the course of the laboratory visit (Table 1). Across the three samples, there was no significant effect of time, indicating that, on average, cortisol concentrations did not change significantly over the visit (*β* = −0.61, *p* = 0.15). Random effects in the model revealed considerable between‐participant variability in baseline cortisol concentrations (*σ* ^2^ = 0.48, 95% CI [0.14, 1.65]; Figure 1). This indicates meaningful individual differences in children's cortisol concentrations at the start of the laboratory visit. A negative covariance between baseline cortisol and the rate of change (*σ* ^2^ = −1.14) suggests that children with higher initial cortisol tended to show steeper declines across the visit.

**Hypothesis 2**. *The child's cortisol response will be associated with length of hospital stay, parental traumatic stress at time of hospitalization for cardiac surgery in infancy, and emotional and behavioral regulation at preschool age*.

Length of hospital stay during infancy (Time 1) was significantly negatively correlated with child cortisol levels at preschool age during the laboratory visit (*ρ* = −0.33, *p* = 0.01), indicating that longer hospital stays following cardiac surgery were associated with lower subsequent cortisol levels. Similarly, parental traumatic stress showed a significant negative correlation with children's average cortisol concentration across the three sampling time points (*ρ* = 0.24, *p* = 0.04, 95% CI [−0.46, −0.002]), indicating that higher parental traumatic stress was associated with lower average child cortisol levels. Emotional and behavioral regulation problems, as measured by CBCL total scores, and internalizing and externalizing subscales were all significantly negatively correlated with average child cortisol levels (*ρ* range: −0.28 to −0.40, *p*s < 0.05). Preschoolers with CHD with emotional and behavioral regulation problems exhibited lower average cortisol levels. Sex differences were observed, with female preschoolers exhibiting lower average cortisol levels than males (*ρ* = −0.45, *p* = 0.02). A significant association was found between relationship quality and average cortisol levels (Mann–Whitney *U* = −2.06, *p* = 0.02; see Hypothesis 3 analyses).

**Hypothesis 3**. The quality of the parent–child relationship will be associated with the cortisol response.

**FIGURE 2:**
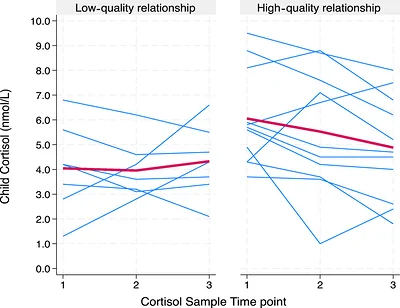
Cortisol mean concentrations and trajectory over time by parent–child relationship quality. Individual salivary cortisol concentrations are shown across the three sample time points, separately for low and high parent–child relationship quality groups. Blue lines represent individual child trajectories, and the red line represents mean cortisol concentration at each time point within each relationship quality group. Cortisol concentrations are reported in nanomoles per liter (nmol/L).

At Time 2, 54% (*n* = 12) of dyads were classified as having high‐quality relationships and 46% (*n* = 10) as having low‐quality relationships (total sample *n* = 22). Analyses examining associations with cortisol concentrations were based on the subset with complete cortisol data (*n* = 19). Visual inspection of cortisol trajectories suggested variability in patterns of change across the laboratory visit according to relationship quality (Figure 2).

### 3.2 Adjusting for Length of Hospital Stay

A mixed‐effects regression model examined the association between relationship quality, time, and child cortisol concentrations, controlling for length of hospital stay and participant‐level variability (Table 2). The main effect of relationship quality on child cortisol concentrations was not significant (*B* = 1.67, *p* = 0.08), although the direction of the association suggested higher cortisol concentrations in children in higher quality relationships. There was some evidence of an interaction between relationship quality and time at Sample 3 (*B* = −1.45, *p* = 0.04). As illustrated in Figure 2, children in higher quality relationships appeared to show a greater reduction in cortisol concentrations across the laboratory visit, although their cortisol levels remained higher overall compared to those in lower quality relationships. Given the small sample size, model complexity, and absence of correction for multiple comparisons, this interaction effect should be interpreted cautiously.

**TABLE 2:** Mixed‐effects model: Parent–child relationship quality, time, and length of hospital stay in relation to child cortisol concentrations.

Variable	*B* (SE)	*z*	*p*	95% CI
Fixed effects				
High‐quality relationship^a^	1.67 (0.98)	1.70	0.08	[−0.25, 3.60]
Sample 2^b^	−0.08 (0.55)	−0.15	0.87	[−1.17, 1.00]
Sample 3^b^	0.28 (0.55)	0.52	0.60	[−0.80, 1.37]
Interaction effects				
High‐quality relationship × Sample 2	−0.44 (0.71)	−0.62	0.53	[−2.82, 1.94]
High‐quality relationship × Sample 3	−1.45 (0.71)	−2.06	0.04^*^	[−2.84, −0.07]
Additional covariates				
Length of hospital stay	−0.02 (0.01)	−1.09	0.28	[−0.05, 0.01]
Constant	4.73 (0.97)	4.88	<0.000^**^	[2.83, 6.64]
Random effects				
Variance (intercept)	2.67			[1.18, 6.03]
Variance (residual)	1.08			[0.66, 1.76]
Model fit				
Log restricted‐likelihood	−95.98			
Likelihood ratio test (vs. linear model)	25.18		<0.001^**^	

*Note: B* = unstandardized regression coefficient.

Abbreviations: CI, confidence interval; SE, standard error.

^a^ Low‐quality relationship is the reference.

^b^ Sample 1 is the reference.

*p < 0.05; **p ≤ 0.001.

There were no significant main effects of time, with cortisol concentrations not differing significantly between Sample 1 and Sample 2 (*B* = −0.09, *p* = 0.87) or between Sample 1 and Sample 3 (*B* = 0.29, *p* = 0.61). Length of hospital stay at Time 1 was not significantly associated with cortisol concentrations (*B* = −0.02, *p* = 0.28). Significant variability in cortisol concentrations was observed between participants (variance = 2.67) and within participants over time (residual variance = 1.08). A likelihood ratio test comparing this model to a simpler model without random effects supported the use of the mixed‐effects model (*χ* ^2^(1) = 25.18, *p* < 0.0001). Sensitivity analyses excluding length of hospital stay as a covariate yielded a comparable pattern of findings, with relationship quality significantly associated with cortisol concentrations in the unadjusted model.

### 3.3 Adjusting for Parental Traumatic Stress

A second mixed‐effects model examined parental traumatic stress as a covariate to assess whether parental traumatic stress uniquely contributed to cortisol levels while accounting for relationship quality and time (Table 3). There was a significant main effect of relationship quality (*B* = 2.33, *p* = 0.01), with higher cortisol concentrations observed in children with higher quality relationships. There was some evidence of a relationship quality and time interaction at Sample 3 (*B* = −1.45, *p* = 0.04). This suggested a greater reduction in cortisol concentrations across the laboratory visit among children with higher quality relationships, although their cortisol concentrations remained higher overall than those in lower quality relationships. As above, this interaction should be interpreted cautiously.

**TABLE 3:** Mixed‐effects model: Parent–child relationship quality, time, and parent traumatic stress in relation to child cortisol concentrations.

Variable	*B* (SE)	*z*	*p*	95% CI
Fixed effects				
High‐quality relationship^a^	2.33 (0.96)	2.43	0.01^*^	[0.45, 4.22]
Sample 2^b^	−0.08 (0.55)	−0.15	0.87	[−1.17, 1.00]
Sample 3^b^	0.28 (0.55)	0.52	0.60	[−0.80, 1.37]
Interaction effects				
High‐quality relationship × Sample 2	−0.44 (0.71)	−0.62	0.53	[−1.83, 0.95]
High‐quality relationship × Sample 3	−1.45 (0.71)	−2.06	0.04^*^	[−2.84, −0.07]
Additional covariates				
Parent traumatic stress	−0.07 (0.05)	−1.26	0.20	[−0.17, 0.04]
Constant	4.45 (0.79)	5.61	<0.000^**^	[2.89, 6.00]
Random effects				
Variance (intercept)	2.59			[1.14, 5.87]
Variance (residual)	1.08			[0.66, 1.76]
Model fit				
Log restricted‐likelihood	−95.98			
Likelihood ratio test (vs. linear model)	24.61		<0.001^**^	

*Note: B* = unstandardized regression coefficient.

Abbreviations: CI, confidence interval; SE, standard error.

^a^ Reference category: Low‐quality relationship.

^b^ Reference category: Sample 1.

* *p* < 0.05;

** *p* ≤ 0.001.

Parent traumatic stress was not significantly associated with child cortisol concentrations in this model (*B* = −0.07, *p* = 0.20).

## 4 Discussion

This study represents an exploratory analysis conducted in a relatively small sample, providing an initial examination of cortisol responding in this population. Findings should be interpreted with appropriate caution, particularly given the number of comparisons examined. Contrary to our expectations, we found no significant overall change in cortisol concentrations across the duration of the visit. This finding aligns with the McGauran et al. (2017) report of a blunted cortisol profile in the home setting. Their study showed lower diurnal cortisol levels at home in children who had early surgery, with a flatter circadian curve and lower overall cortisol. These samples, collected on weekends and thus less influenced by weekday routines, further highlight the potential for sustained alterations in stress physiology. Indeed, traumatic, uncontrollable, or life‐threatening stressors have been shown to lead to acute elevations in cortisol levels soon after the stressor but, with time and chronicity, elicit a flat pattern of cortisol production across the day (Hostinar and Gunnar 2013). Viewed together, these results suggest that children with early cardiac surgery histories may exhibit altered stress regulation in both medical and everyday settings, though the specific context and type of stressor may differentially shape children's physiological responses.

Children with greater emotional and behavioral difficulties at preschool age showed lower cortisol levels during the laboratory visit. Similar patterns have been observed with internalizing symptoms early in life (McGinnis et al. 2016) and externalizing behaviors in adolescence (Platje et al. 2013) linked to blunted cortisol response. Interestingly, higher parental traumatic stress at the time of surgery was associated with lower preschool child cortisol levels. However, when relationship quality was accounted for, this association was no longer significant. This suggests that high‐quality parent–child relationships may buffer the impact of initial parental distress on children's stress physiology. When parents experience trauma‐related distress, their capacity to provide emotionally available caregiving may be diminished. Parental buffering is critical for regulating infants’ stress responses during early development (Gunnar and Donzella 2002; Hostinar et al. 2015) and during physically painful events (Gunnar et al. 1996). When this co‐regulation is compromised, either by parental distress or by the intensity of medical demands, the infant's developing stress system may adapt by downregulating HPA axis activity, leading to lower cortisol levels that persist into later childhood.

Children in less emotionally available relationships exhibited relatively stable and lower cortisol levels across sampling time points, a pattern consistent with hypoarousal or blunted HPA axis activity. Such relationships might not provide attuned, responsive buffering to support the child's reactivity and recovery during a stressful experience. This pattern aligns with previous research demonstrating that children exposed to stressful experiences in the absence of co‐regulation and buffering support can develop reduced cortisol production over time (Hostinar and Gunnar 2013; McLaughlin et al. 2015). Sturge‐Apple et al. (2012) suggest that blunted cortisol responses may be indicative of children attempting to downregulate the psychological impact and provide a temporary means of attaining a perceived sense of security or control in stressful contexts.

An additional consideration is whether the laboratory visit was experienced as stressful, particularly in the absence of a medical procedure. Children and their parents may have also developed a degree of familiarity or habituation to medical environments over time, given repeated exposure to hospital settings for surgery, treatment, and follow‐up care. Consistent with this, repeated exposure to stressors has been associated with attenuated cortisol response (Buske‐Kirschbaum et al. 1997; Gunnar and Quevedo 2007), which may reduce physiological reactivity in this context even when underlying vulnerabilities in stress regulation remain. However, McGauran et al. (2017) demonstrated differences in cortisol responding during medical procedures, including ECG assessment, among children with CHD and early surgical exposure, suggesting that medical contexts may still elicit physiological differences in this population.

Repeated or sustained exposure to stress is proposed to lead to physiological adaptations of the HPA axis, specifically to attenuated cortisol responses over time (Koss and Gunnar 2018; McEwen 1998). Notably, our results showed that longer hospital stays, representing prolonged stress exposure, were associated with lower cortisol levels at preschool age. Similar findings have been reported in other pediatric populations, such as children with atopic dermatitis (Buske‐Kirschbaum et al. 1997) and children exposed to early adversity (Bunea et al. 2017). Importantly, this research also contributes to the broader study of early life stress. CHD provides a form of natural experiment in which children are exposed to well‐defined and time‐specific stressors in infancy, without the confounding influences commonly found in high‐risk samples, such as those experiencing socioeconomic adversity (Gunnar and Quevedo 2007). Therefore, the findings have relevance beyond CHD, offering insight into mechanisms shaping the development of the HPA axis in early childhood.

These interpretations highlight the importance of considering both the nature of the visit and the child's prior experiences when interpreting patterns of cortisol responding. Together, these findings suggest that both situational factors (e.g., familiarity with the medical context) and longer‐term adaptations in stress physiology may contribute to the patterns observed.

Findings from the mixed‐effects models provide tentative insight into the relationship between parent–child relationship quality and children's cortisol response. Across models, children in higher‐quality relationships showed higher cortisol concentrations overall, although this association was not statistically significant in all models. There was also some evidence that children in emotionally available relationships showed an initial rise in cortisol, followed by a decline, possibly consistent with an adaptive mobilization of physiological resources and subsequent recovery (Dickerson and Kemeny 2004). This activation enables children to remain alert and responsive to their surroundings, which is particularly relevant in settings requiring medical intervention. While we did not capture the full trajectory of recovery, the observed patterns show emotionally available parents help children engage with stressors in a way that supports adaptive coping. Although preliminary, the decrease in cortisol levels at the third sample suggests that the buffering effect of relationship quality might be more apparent later in the hospital visit. This is consistent with a recent study demonstrating that higher quality maternal caregiving was associated with lower levels of infant cortisol at the end of a laboratory visit and less infant crying (Bruinhof et al. 2025). Indeed, higher quality maternal caregiving is related to quicker cortisol recovery from stress responses, and infants with less sensitive, more intrusive mothers have been shown to retain higher cortisol concentrations for a longer period of time after a stressful event (Albers et al. 2008). Clinically, these findings tentatively highlight the potential importance of fostering positive parent–child emotional availability and sustaining parental support throughout stressful experiences, such as medical visits, to help regulate children's stress over time.

### 4.1 Limitations

Although our study offers valuable insights into the association between parent–child relationship quality and cortisol levels, the design did not allow for capturing additional cortisol samples to better understand the recovery phase of the cortisol response. In addition, the absence of a nonhospital baseline measure means that changes in cortisol observed during the laboratory visit cannot be definitively attributed to the visit as a stressor, independent of typical diurnal variation. A longer visit, incorporating additional saliva samples over time, may have revealed differences in cortisol recovery that were not detectable within our limited time frame. Given the challenges of requiring preschool‐aged children to attend lengthy appointments, particularly given health vulnerabilities associated with CHD, future studies could explore alternative, child‐friendly approaches. Methods such as incorporating cortisol sampling during routine clinical visits or home‐based sampling using interactive virtual tasks may overcome logistical and health‐related constraints, while providing a comprehensive picture of cortisol dynamics in everyday experiences. These methods may offer a feasible and ethically appropriate balance between robust data collection and the practical realities of conducting research with young children experiencing medical adversity.

The modest sample size limited statistical power, particularly in models including multiple predictors and interaction terms, and may have increased the influence of individual observations on effect estimates and tests of statistical significance. Accordingly, findings should be interpreted cautiously and considered exploratory.

A further limitation is the time elapsed between the assessment of early experiences in infancy and outcomes measured at preschool age. Changes in the caregiving environment, parental well‐being, and broader contextual factors over this period were not fully captured and may have influenced child outcomes. Although a range of psychosocial variables were collected at Time 1, including parental mental health, only a subset were included in the present analyses. In addition, length of stay was used as a proxy for early medical stress; however, this objective indicator may not reflect families’ subjective experience of stress during hospitalization. Future research incorporating both objective and subjective measures of stress across development would provide a more comprehensive understanding of these processes.

Qualitative insights from children and their parents may further help to contextualize cortisol patterns and identify stressors, buffers, or coping mechanisms that are not captured in quantitative measures alone. Such mixed‐methods approaches would strengthen the interpretation of biological data and may guide tailored psychosocial interventions. Future studies with larger sample sizes are needed to increase statistical power to detect subtle but clinically meaningful changes in cortisol responses. The considerable individual variability observed in cortisol levels across participants may reflect both biological differences in cortisol reactivity and unmeasured contextual influences. This variability, while expected in studies of this nature, may have obscured subtle (sub)group‐level patterns.

### 4.2 Overall, Our Results Reinforce the Importance of Early Experiences

This study extends previous research by indicating that surgical and medical experiences in infancy may have a lasting impact on cortisol response in children with CHD. Importantly, high‐quality parent–child relationships, characterized by emotional availability, had a meaningful impact by buffering children's cortisol response. The associations between length of hospital stay, parental traumatic stress, and lower cortisol responses underscore the need for early psychosocial support for families undergoing prolonged or intensive medical treatment early in life. Our findings align with the toxic stress model (Shonkoff and Garner 2012), providing further evidence that social buffers in the context of early adversity (e.g., cardiac surgery for CHD) may help shift a child's response from toxic to tolerable stress (e.g., indexed by cortisol). Our results highlight the key role of parent–child relationships in facilitating adaptive physiological response to stress in the context of medical adversity. Indeed, interventions aimed at improving relationships or reducing psychosocial stressors have been shown to normalize cortisol production, suggesting that the stress response system is adaptable and can be positively influenced by supportive care (Slopen et al. 2014). Future studies should investigate how interventions that promote emotionally available relationships during and after cardiac surgery may influence children's stress regulation over time.

## Funding

Tamera Clancy was supported by an Australian Government Research Training Program Scholarship.

## Ethics Statement

The Human Research and Ethics Committee (HREC) at the Royal Children's Hospital (RCH), Melbourne, Australia, approved all methods and procedures used in this study, and all parents gave written informed consent prior to participation (HREC # 34174).

## Conflicts of Interest

The authors declare no conflicts of interest.

## Supporting information




**Supplementary Material**: dev70200‐sup‐0001‐TableS1.docx

**Supplementary Material**: dev70200‐sup‐0002‐TableS2.docx

## Data Availability

The data that support the findings of this study are available on request from the corresponding author. The data are not publicly available due to privacy or ethical restrictions.
