# Supplementary material for: Early Childhood Emotional, Relational, and Stress‐Related Outcomes Following Infant Cardiac Surgery
Source: Dev Psychobiol. 2026 Sep 14;68(5):e70200. doi: 10.1002/dev.70200 (PMC13575582; doi:10.1002/dev.70200)
Supplement: Supplementary file 1 [file DEV-68-e70200-s001.docx]

Supplementary Table 1.

Laboratory Visit Schedule and Saliva Sampling Timeline

| **Visit Phase** | **Activities** | **Approximate duration** | **Saliva sampling** | **Timing reference**  **(approximately)** |
| --- | --- | --- | --- | --- |
| **Arrival & familiarisation** | Welcome, free play, orientation | 10 min | S1 | 10 min after arrival |
| **Structured tasks** | Stranger Approach; Attractive toy in a transparent box; Gift Wrap/Delay | 25 min | S2 | 25−30 min after S1 (*M* = 24 min) |
| **Parent-child interaction** | Free play with standardised toys | 20 min | S3 | 50−60 min after S1 (*M* = 51 min) |

*Note:* Durations are reported in minutes (min). *M* = mean. Parent-child interaction was designed to last 20 minutes. Observed duration across dyads was *M* = 19.77 min (SD = 2.11, range = 15.08 - 23.72).
