# Supplementary material for: Early Childhood Emotional, Relational, and Stress‐Related Outcomes Following Infant Cardiac Surgery
Source: Dev Psychobiol. 2026 Sep 14;68(5):e70200. doi: 10.1002/dev.70200 (PMC13575582; doi:10.1002/dev.70200)
Supplement: Supplementary file 2 [file DEV-68-e70200-s002.docx]

Supplementary Table 2.

Brief description of Emotional Availability (EA^®^) Dimensions, adapted from Biringen (2008)

| **Scale** | **Description** |
| --- | --- |
| *Parent Sensitivity* | The parent’s ability to perceive, interpret and appropriately respond to their child’s cues and emotions during interactions. It encompasses the expression of positive affect, acceptance of the child’s behaviour, flexibility, ability to handle conflicts, and sensitivity to timing. |
| *Parent Structuring* | The parent’s capacity to scaffold the child’s behaviour in a developmentally appropriate way and to guide and support the child while following the child’s lead. This includes setting appropriate limits, establishing rules and using verbal and non-verbal strategies to promote cooperation and regulation. |
| *Parent Non-intrusiveness* | The parent’s ability to support the child’s autonomy without interfering inappropriately. This is reflected in the absence of over-direction, over-stimulation or interferences. |
| *Parent Non-hostility* | The absence of hostile, impatient, rejecting or harsh behaviour, including covert or overt negative affect toward the child. |
| *Child Responsiveness* | The child’s affective and behavioural responsiveness to the adult’s cues. This includes age-appropriate autonomy-seeking, physical proximity, interest in the interaction, and the absence of role-reversal, avoidance or disengagement. |
| *Child Involvement* | This child’s active engagement with and involvement of the adult during interaction. This includes the child’s ability to initiate contact, use the adult for emotional or instrumental support, and maintain connection through eye contact, body positioning, and verbal interaction. |
